# Preliminary Results of Hyperthermic Intraperitoneal Intraoperative Chemotherapy as an Adjuvant in Resectable Pancreatic Cancer

**DOI:** 10.1155/2012/506571

**Published:** 2012-05-27

**Authors:** Antonios-Apostolos K. Tentes, Dimitrios Kyziridis, Stylianos Kakolyris, Nicolaos Pallas, Georgios Zorbas, Odysseas Korakianitis, Christos Mavroudis, Nicolaos Courcoutsakis, Panos Prasopoulos

**Affiliations:** ^1^Surgical Department, Didimotichon General Hospital, Didimotichon 68300, Greece; ^2^Department of Clinical Oncology, Alexandroupolis General Hospital, Democritus University of Thrace, Greece; ^3^Department of Anesthesiology, Didimotichon General Hospital, Greece; ^4^Department of Radiology, Alexandroupolis General Hospital, Democritus University of Thrace, Greece

## Abstract

*Background and Aims*. 5-year survival in patients with pancreatic cancer is poor. Surgical resection is the only potentially curative resection. The results of adjuvant treatment either with chemotherapy or with radiotherapy have been contradictory and the incidence of local-regional recurrence remains high. If local-regional recurrence is controlled survival may be expected to increase. Hyperthermic intraoperative intraperitoneal chemotherapy (HIPEC) may be used in order to control local-regional recurrences. The purpose of the study is to identify the effect of HIPEC in patients with pancreatic cancer undergoing potentially resection. *Patients and Methods*. From 2007–2011, 21 patients, mean age 69.4 ± 9.5 (50–86) years, underwent tumor resection, and HIPEC with gemcitabine. The hospital mortality and morbidity rate was 9.5% and 33.3%, respectively. 5-year and median survival was 23% and 11 months, respectively. The recurrence rate was 50% but no patient developed local-regional recurrence. No patient was recorded with gemcitabine-induced toxicity. *Conclusions*. This clinical study of 21 patients is the first to combine an R_0_ pancreas cancer resection with HIPEC. Increased morbidity and mortality from intraoperative gemcitabine was not apparent. Patients with pancreatic cancer undergoing potentially curative resection in combination with HIPEC may be offered a survival benefit. Data suggested that local-regional recurrences may be greatly reduced. Further studies with greater number of patients are required to confirm these findings.

## 1. Introduction

Pancreatic cancer is one of the most frequent causes of cancer-related deaths in the western world. The overall 5-year survival rate after potentially curative resection does not exceed 15% in most series [1–3], although in high volume centers it may be as high as 20–25% [4, 5]. Surgical resection remains the single potentially curative option but only 10–15% of the diagnosed tumors are eligible for resection [6–9]. Increase of long-term survival may result either if the proportion of patients with locally unresectable tumors decreases or if treatments that may control disease recurrence, and particularly the local-regional ones, are developed.

In 1985 the Gastrointestinal Study Group showed that adjuvant chemoradiation offers significant survival benefit after surgical resection in patients with pancreatic cancer [10] but a decade later this was disputed by the study conducted by EORTC [11]. The ESPAC study showed that chemotherapy only offers a survival benefit [12]. Recent studies have shown that chemoradiation may be a favorable option for patients with resectable tumors [13]. A review of these manuscripts document that the data concerning adjuvant treatment for resectable pancreatic cancer are contradictory.

The sites of recurrence after curative resection are the liver in 50–60%, the peritoneal surfaces in 40–50%, and the pancreatic bed in 50% of the cases [14]. The pathophysiology of local-regional recurrence after R_0_ resection remains an enigma. It may be the result of metastases undetected on imaging or laparotomy. Or tumor dissemination and implantation of cancer emboli at the resection sites may occur with pancreatectomy [15]. If this is true then intraperitoneal chemotherapy may be the treatment that has a beneficial impact on overall survival by reducing the number of local-regional recurrences. Intraperitoneal chemotherapy has the capability to eradicate the microscopic cancer emboli and reduce the incidence of local-regional recurrences. It is obvious that there is an absolute need for adjuvant treatment in addition to surgical resection.

The purpose of the study is to identify the potential benefits of hyperthermic intraperitoneal intraoperative chemotherapy (HIPEC) with gemcitabine in patients that undergo R_0_ resection for pancreatic cancer.

## 2. Patients-Methods

From April 2007 until August 2011, 21 patients with resectable pancreatic cancer, without distant metastatic lesions as assessed by routine preoperative staging (physical examination, CT-scan, MRI, and bone scanning) were enrolled in the study. The study was approved by the Ethical Committee of the hospital and patients signed an informed consent prior to accepting this therapeutic approach.

The diagnosis was possible by physical examination, hematological-biochemical examination, tumor markers (CEA, CA 19-9, CA-125), CT abdominal and thoracic scan or MRI, and bone scanning. No preoperative histological examination was performed.

Patients between 16–90 years of age, with satisfactory cardiopulmonary function, satisfactory renal function (blood urea level <50 mg/dL and creatinine level <1.5 mg/dL), satisfactory liver function (other than hepatobiliary obstruction), with white blood cell count >4000/mL, platelet count >150.000/mL, and acceptable performance status (Karnofsky performance status >50%) were included in the study.

Patients with evidence of distant metastatic disease (liver, osseous, brain and pulmonary), with prior antitumor therapy, with prior malignancy at risk for recurrence (except for basal cell carcinoma or in situ carcinoma of the cervix adequately treated), with poor performance status (Karnofsky performance status <50%), with psychiatric diseases or addictive disorders, and pregnant women were not included in the study.

Patients with periampullary tumors were not included in the study. Patients with resectable pancreatic cancer and limited peritoneal metastases for whom CC-0 or CC-1 cytoreduction could be possible, were included in the study.

### 2.1. Treatments

Patients with cancer of the head of the pancreas underwent subtotal pancreatoduodenectomy (Kausch-Whipple procedure). Distal pancreatectomy was used for cancer of the body or the tail of the pancreas. After tumor resection and before the reconstruction of the alimentary tract, HIPEC was performed for 60 min at 42–43°C with gemcitabine at a dose of 1000 mg/m^2^. HIPEC was administered using the open (Coliseum) technique. A heater circulator with two roller pumps, one heat exchanger, one reservoir, and an extracorporeal system of two inflow and two outflow tubes, and 4 thermal probes was used for HIPEC (Sun Chip, Gamida Tech, France). A prime solution of 2-3 liters of normal saline was instilled prior to administration of the cytostatic drug and as soon as the mean abdominal temperature reached 40°C gemcitabine was instilled in the abdomen.

During perfusion adequate fluids were administered in addition to dopamine at a diuretic dose of 3 *μ*g/K.b.w., in order to maintain diuresis at 500 mL/h. Dopamine was also used after surgery for 24 hours to maintain diuresis at the same levels. 

The reconstruction of the alimentary tract was performed after the completion of HIPEC. After subtotal pancreatoduodenectomy the reconstruction was always made with an end-to-side pancreato-jejunal anastomosis, end-to-side choledocho-jejunal anastomosis, followed by a Roux-en-Y gastrointestinal anastomosis with a second jejunal loop.

Cytoreductive surgery with standard peritonectomy procedures was used for the treatment of peritoneal metastases whenever they were found [16]. A CC-0 operation did not leave behind macroscopically visible tumor. A CC-1 operation had residual tumor less than 2.5 mm in its largest diameter [17].

All resected specimens were sent for histopathological examination and complete staging. Stage III patients received additional systemic chemotherapy with gemcitabine and 5-FU.

### 2.2. Followup

All patients were followed up at 3-month intervals with physical examination, hematological, and biochemical examinations, tumor markers (CEA, CA 19-9, CA-125), and thoracic and abdominal CT. Recurrences and the sites of recurrence were recorded.

### 2.3. Statistical Analysis

The proportion of patients with a given characteristic was compared by chi-square analysis or by Pearson's test. Differences in the means of continuous measurement were tested by the Student's *t*-test. The survival curves were obtained with the Kaplan-Meier method. A two-tailed *P* value of <0.05 was considered statistically significant.

## 3. Results

The mean age of the patients was 69.4 ± 9.5 (50–86) years. The characteristics of the patients are listed in Table 1. Histopathology revealed that all patients had pancreatic cancer. One patient with cancer of the pancreatic tail and extensive peritoneal carcinomatosis underwent distal pancreatectomy and near complete cytoreduction (CC-1) combined with HIPEC. This was defined as R_1_ surgery because of possible residual tumor <2.5 mm left on the peritoneal surfaces of the mesentery. All the other patients had resectable tumors and underwent R_0_ resection of the tumor combined with HIPEC. Seventeen patients with tumor of the head of the pancreas underwent subtotal pancreatoduodenectomy. The other four patients (three with cancer of the tail and one with cancer of the body) underwent distal pancreatectomy.

The hospital morbidity rate was 33.3% (7 patients). The recorded complications are listed in Table 2. One patient was reoperated because of postoperative bleeding that was successfully controlled. One further patient was reoperated because the choledochojejunal anastomosis failed, but was successfully controlled by T-tube insertion. The other patient with anastomotic leak underwent conservative treatment. The rate of reoperation was 9.5%. Only one patient was recorded with grade II neutropenia that did not require specific treatment. The hospital mortality rate was 9.5% (2 patients). One of them died because of ARDS and the other one of sepsis with an unknown primary site. The mean hospital length of stay was 18 days.

**Figure 1:**
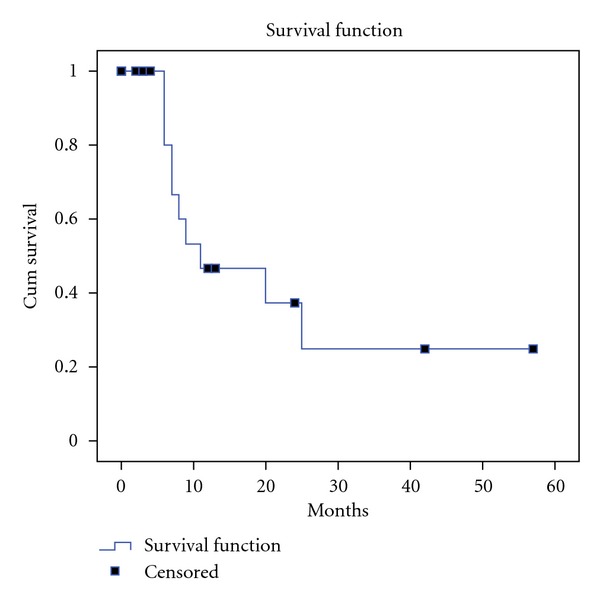
Overall survival of 21 patients with pancreatic cancer treated with complete resection plus hyperthermic intraoperative intraperitoneal chemotherapy.

The 5-year survival rate was 23% and the median survival 11 months (Figure 1). Eleven stage III patients received systemic adjuvant chemotherapy with gemcitabine. One of the patients with stage II disease died during the immediate postoperative period. The median disease-free survival time was 5 months. The median follow-up time was 7 months. During followup 9 patients (50%) were recorded with recurrence. Three of them were stage II and 6 were stage III. All these patients had liver metastases and no locoregional recurrence, was recorded.

Currently 8 patients (38.1%) are alive without evidence of disease, 10 patients (47.6%) died because of recurrence, and 3 patients (14.3%) died of other causes unrelated to cancer.

## 4. Discussion

Although the pathophysiology of local-regional recurrence is unclear it has been assumed that the resection of a tumor located within narrow margins of resection may result in tumor dissemination because of interstitial tissue trauma, or severed lymphatics leaking cancer cells, or from venous blood loss contaminated by cancer cells. The disseminated cancer emboli are trapped in fibrin, stimulated by growth factors, and give rise to local-regional recurrent tumors within months-years after initial surgical manipulations [15]. The eradication of the entrapped microscopic cancer emboli may be possible by using intraperitoneal chemotherapy. Intraperitoneal chemotherapy has been shown to be very effective in carcinomatosis from colorectal cancer either as HIPEC or as early postoperative intraperitoneal chemotherapy (EPIC) under normothermia. The advantage of intraperitoneal chemotherapy is the high drug level that can be achieved by low systemic exposure [18].

Gemcitabine as systemic adjuvant treatment has been proved to be very effective in high risk patients undergoing potentially curative resection [19]. However, systemic chemotherapy has not been confirmed to assist in control of local disease. In contrast, it has been shown both from laboratory and clinical studies that the intraperitoneal use of gemcitabine may effectively target local disease. Laboratory studies have shown that the intraoperative use of gemcitabine may effectively prevent the development of peritoneal metastases. In addition early postoperative intraperitoneal chemotherapy may reduce the extent of peritoneal metastases [20]. Our data shows that the intraperitoneal use of gemcitabine in patients having pancreatectomy is well tolerated and does not produce severe toxicity. After all, only one patient developed grade II neutropenia that did not require any specific treatment. Intraperitoneal gemcitabine may be incriminated for the two anastomotic failures although it has not been proved. The large concentration of gemcitabine sustained in the peritoneal space and the low plasma concentration are findings supporting its intraperitoneal use [21].

The theoretical advantage of intraperitoneal gemcitabine has been confirmed by clinical and laboratory studies. Pharmacokinetic studies of intraperitoneal administration in a rat model have demonstrated that the area under the curve ratio of intraperitoneal to systemic drug exposure is closely related to the intraperitoneal dose and tissue samples showed increased drug concentration when administered with heat [22]. Preliminary pharmacokinetic data in patients with resectable pancreatic cancer that underwent HIPEC with gemcitabine at a dose of 1000 mg/m^2^ showed marked local-regional drug exposure [23]. In addition, the intraperitoneal use of gemcitabine in clinical practice has shown equal results to platinum-based regimens in women with ovarian cancer [24]. These data taken together suggest that studies to test gemcitabine in patients with resectable pancreatic cancer are justified. It appears that intraperitoneal chemotherapy may have a favorable effect in eradicating microscopic cancer emboli not only locoregionally but also in the portal venous circulation. It has been found that the measured portal vein concentrations exceeded the measured concentration in other vessels when 5-FU was administered intraperitoneally [25]. Although the number of the included patients is very small and the median follow-up time short, no patient developed local-regional recurrence. This implies that HIPEC is likely to be effective in eradicating residual microscopic cancer emboli at the peritoneal surfaces.

## 5. Conclusions

Our preliminary results in the resection of pancreatic cancer with HIPEC using gemcitabine have shown that there may be a survival advantage even in patients with nodal involvement.

## Figures and Tables

**Table 1:** Patients' general characteristics.

Male/Female	No. of patients	%
	9/12	42.9/57.1
Tumor anatomic distribution		
Head	17	81
Body	1	4.8
Tail	3	14.3

Performance status		
90–100%	15	71.4
70–80%	5	23.8
50–60%	1	4.8

Tumor infiltration		
T_1_	1	4.8
T_2 _	3	14.3
T_3_	17	81

Nodal infiltration		
N_0_	9	42.9
N_1_	12	57.1

TNM stage		
I	3	14.3
II	6	28.6
III	12	57.1

Degree of differentiation		
G_1_	4	19
G_2_	9	42.9
G_3_	8	38.1

Residual tumor		
R_0_	20	95.3
R_1_	1	4.7

**Table 2:** Postoperative complications.

	No. of patients	%
Postoperative bleeding	1	4.8
Anastomotic leak	2	9.5
Acute respiratory distress syndrome	2	9.5
Sepsis	1	4.8
Grade II neutropenia	1	4.8
